# Task-Sharing of HIV Care and ART Initiation: Evaluation of a Mixed-Care Non-Physician Provider Model for ART Delivery in Rural Malawi

**DOI:** 10.1371/journal.pone.0074090

**Published:** 2013-09-16

**Authors:** Megan McGuire, Jihane Ben Farhat, Gaelle Pedrono, Elisabeth Szumilin, Annette Heinzelmann, Yamikani Ntakwile Chinyumba, Sylvie Goossens, Simon Makombe, Mar Pujades-Rodríguez

**Affiliations:** 1 Epicentre, Paris, France; 2 Médecins sans Frontières, Medical Unit, New York, New York, United States of America; 3 Médecins sans Frontières, Chiradzulu, Malawi; 4 Médecins sans Frontières, Paris, France; 5 HIV Unit, Ministry of Health, Lilongwe, Lilongwe, Malawi; 6 University College London, London, United Kingdom; Vanderbilt University, United States of America

## Abstract

**Background:**

Expanding access to antiretroviral therapy (ART) in sub-Saharan Africa requires implementation of alternative care delivery models to traditional physician-centered approaches. This longitudinal analysis compares outcomes of patients initiated on antiretroviral therapy (ART) by non-physician and physician providers.

**Methods:**

Adults (≥15 years) initiating ART between September 2007 and March 2010, and with >1 follow-up visit were included and classified according to the proportion of clinical visits performed by nurses or by clinical officers (≥80% of visits). Multivariable Poisson models were used to compare 2-year program attrition (mortality and lost to follow-up) and mortality by type of provider. In sensitivity analyses only patients with less severe disease were included.

**Results:**

A total of 10,112 patients contributed 14,012 person-years to the analysis: 3386 (33.5%) in the clinical officer group, 1901 (18.8%) in the nurse care group and 4825 (47.7%) in the mixed care group. Overall 2-year program retention was 81.8%. Attrition was lower in the mixed care and higher in the clinical officer group, compared to the nurse group (adjusted incidence rate ratio [aIRR]=0.54, 95%CI 0.45-0.65; and aIRR=3.03, 95%CI 2.56-3.59, respectively). While patients initiated on ART by clinical officers in the mixed care group had lower attrition (aIRR=0.36, 95%CI 0.29-0.44) than those in the overall nurse care group; no differences in attrition were found between patients initiated on ART by nurses in the mixed care group and those included in the nurse group (aIRR=1.18, 95%CI 0.95-1.47). Two-year mortality estimates were aIRR=0.72, 95%CI 0.49-1.09 and aIRR=5.04, 95%CI 3.56-7.15, respectively. Slightly higher estimates were observed when analyses were restricted to patients with less severe disease.

**Conclusion:**

The findings of this study support the use of a mixed care model with well trained and regularly supervised nurses and medical assistants to provide HIV care in countries with high HIV prevalence.

## Introduction

Despite the unprecedented global scale-up of access to antiretroviral therapy (ART), almost half of all people living with HIV still do not have access to treatment [1]. Difficulties in expanding access are related to many factors, including remoteness of health facilities, unstable program financing, complex drug supply and storage systems and poor individual awareness of HIV status [2]. Another limitation is an acute shortage of health care workers [3-7]. While sub-Saharan Africa bears the heaviest HIV disease burden globally, it hosts only 3% of trained health care workers [8].

Physician- and hospital-centered care delivery approaches have not been brought to scale in many countries throughout sub-Saharan Africa due to limited number of doctors, overwhelmed and overflowing clinics, and to the distances that many patients must travel to reach these centralized facilities [9,10]. This shortage of traditionally utilized health care workers, such as doctors, coupled with limited simplified care delivery models has hindered efforts to reach patients in urgent need of treatment [11,12].

At the end of 2009 only 64% of countries in sub-Saharan Africa had developed policies to address human resource shortages through task-shifting strategies [13]. Too few countries have implemented clear policies defining task-shifting roles, responsibilities and limits [14]. While there is increasing evidence that non-physician HIV care delivery is feasible, the extent to which task-shifting approach is viable without reducing quality of care is unknown, especially in large decentralized HIV programs [4,15-18]. In a systematic review, Callaghan and colleagues reported task-shifting from doctors to nurses to be effective, but highlighted that implementation is challenging, and that there is a need to ensure adequate training and supervision of staff [19]. However, there are few patient outcome data from real-life settings where nurses are responsible for ART initiation and follow-up [20]. Furthermore available reports might not reflect what is feasible within the constraints of rural, decentralized settings with large numbers of patients.

With one of the highest HIV burdens in sub-Saharan Africa but just 2 physicians, 7 clinical officers, 7 medical assistants and 37 nurses per 100,000 individuals, Malawi is facing a severe human resources crisis [21]. In this context, utilization of all cadres of health workers and of alternatives models of care are crucial for expanding ART delivery to all eligible patients. In 2007, the Malawian Ministry of Health revised its policies to allow nurses and medical assistants to initiate and prescribe ART [7]. At that time, Médecins Sans Frontières (MSF) developed an ART delivery training and implementation strategy for nurses and medical assistants to scale up treatment in the rural district of Chiradzulu. As this new approach was implemented and decentralized health centers had daily HIV clinics, annual ART initiations went from 2,316 in 2006 to 4,194 in 2008. At the end of 2010 over 26,000 patients had been initiated on treatment [22].

In this cohort study we assess 2-year program retention and patient mortality among 10,000 adult patients receiving ART in the Chiradzulu program, comparing patients monitored by nurses or medical assistants to those followed by clinical officers.

## Methods

### Study Setting

Since 2001, MSF, in collaboration with the Malawian Ministry of Health, has provided free HIV/AIDS care, including ART, in the Chiradzulu District [22,23]. This district has an estimated population of 310,000 individuals and 20% HIV prevalence. Care is delivered both at the district hospital and at 10 decentralized rural health facilities. Prior to September 2006, clinical officers were the primary care providers (ART initiation and follow-up) while nurses performed other tasks such as drug dispensing, adherence assessment, blood draw, and registration.

Extensive scale-up of HIV services through decentralized health facilities required reorganization and redistribution of responsibilities to non-physician or non-nurse health workers, and additional training of these providers (Table S1). Nurse-targeted training modules commenced in 2005, followed by a 2-month practical assessment. Nurses began following-up stable patients in September 2006 and initiating patients on ART in May 2007. Ongoing assessment of nurses’ competencies is routinely performed through supervision focused on the ability to correctly stage patients, diagnose simple opportunistic infections, interpret CD4 count measurements; assess patient readiness to start ART, appropriately referral of complicated cases and utilization of monitoring tools (Table S2).

Registration, basic triage and contact tracing within 1-2 days of missed appointments (for patients who lived within the boundaries of the district) are conducted by community health workers. The program utilized peer counselors living with HIV to delivery adherence support. These counsellors are trained through practical/problem-based modules covering patient education, psychosocial support and assessment of ARV readiness and follow-up ARV counseling. No national physicians contributed to routine care management in Chiradzulu, although 2-3 international physicians provided clinical management support in the program.

### Patient Management

Criteria for ART initiation were based on national treatment guidelines: clinical stage 3 or 4, or a CD4 cell count <250 cells/µL. Clinical appointments were scheduled at a maximum of once every 3 months. Individual and group counseling, including adherence support, was provided by trained counselors and peer workers. CD4 count testing was monitored before and every year after starting ART. First and second-line ART regimens were non-nucleoside containing regimens and protease-inhibitor based therapy, respectively. Viral load testing was performed only when treatment failure was suspected based on clinical or immunological deterioration.

Nurses and medical assistants primarily managed less complicated patients: CD4 cell count >100 cells/µL, clinical stage 1 or 2, BMI >18 kg/m^2^ and receiving first line ART. Clinical officers and physicians managed both complicated and uncomplicated patients. Complicated cases were defined as patients with suspicion of tuberculosis or treatment failure, diagnosed with Kaposi’s sarcoma or receiving second line ART, pregnant women or age <13 years old. Patients followed by nurses and presenting with complications at follow-up visits were referred to clinical officers, while patients followed by clinical officers were eligible for nurse-provided care after clinical stabilization.

Socio-demographic and clinico-immunological data, treatment information and health care provider during consultation were routinely recorded on medical files and entered into an electronic database (FUCHIA; Epicentre, Paris, France). Standard data consistency checks and verifications were performed routinely both on and off site.

### Study population and definitions

All adult patients (≥15 years) who started ART in the Chiradzulu HIV program between September 2007 and March 2010 and with more than one follow-up visit after ART initiation were included in the analysis. Patients cared by nurses or medical assistants for ≥80% of their clinical visits were included in the “nurse group”, those cared by clinical officers or physicians for ≥80% of visits were included in the “clinical officer group”, and those with <80% of visits by the same type of provider were categorized as “mixed care group”. For secondary analyses, mixed care group patients were categorized into 2 subgroups according to the type of provider who prescribed ART at the date of therapy start (clinical officer/physician or nurse/medical assistant).

### Statistical Analysis

Patient baseline characteristics and absolute CD4 cell count gains were described by provider group using summary statistics (proportions and medians with interquartile ranges).

Patient follow-up was right-censored at the earliest of time of death, transfer outside the program, last clinical visit, or 24 months after ART start. Kaplan-Meier survival techniques allowed estimating cumulative probabilities of program attrition and mortality by type of provider 3, 6 and 24 months after ART start.

Multivariable Poisson models were used to examine the association between 2-year program attrition or mortality and type of provider, initially treated as a 3-group categorical variable (nurse group, mixed care group, and clinical officer group). Crude and adjusted incidence rate ratios (IRR) with 95% confidence intervals (CI) were reported. Factors considered in the analysis included: sex, initial BMI (≤18.5, 18.5-24.99, ≥25 and missing), clinical stage (1, 2, 3, 4 and missing) and CD4 cell count (<50, 50-99, 100-199, 200-249, ≥250 cells/µL and missing); year of ART initiation (2007, 2008, 2009 and 2010), adherence index (≥95%, 80-94% and <80%), and patient follow-up split into 3 periods (≤3, >3-6 and >6-24 months). The proxy for adherence was based on calculation of the incidence of missed clinical appointments during study follow-up (<5% corresponding to >95% adherence, 5-9.99% to 90-95%, and ≥10% to <90% adherence [24]. All individual-level factors associated with the outcome (*P*<0.2) in univariable analysis were included in the final models. The likelihood ratio test for association was used and the level of statistical significance considered was *P*<0.05. The fit of the final models was assessed with the goodness-of-fit test for Poisson. In secondary analyses, to examine the influence of the type of provider who initiated ART on the outcomes, analyses were carried out using the 4-group categorical type of provider variable (nurse, mixed care nurse initiated, mixed care clinical officer initiated, and clinical officer categories).

Sensitivity analyses were performed by repeating the analyses in the subgroup of patients with less severe HIV disease at ART start, defined as: BMI≥18.5 kg/m^2^, a clinical stage 1 or 2 and a CD4 count ≥100 cells/µL (definition of uncomplicated cases in the HIV program). Analyses of complete case data (excluding patients with missing CD4 count and clinical stage information) were also performed. In addition, analyses were repeated in the subgroup of patients who were in care after 3 months of ART use. Data were analyzed using Stata 11 (Stata Corp, College Station, Texas, USA).

### Ethics Statement

In agreement with the Ministry of Health of Malawi, medical patient information is prospectively collected in the Chiradzulu HIV program for evaluation and to facilitate patient medical monitoring. This information is collected without obtaining prior written informed consent from patients or the guardians of minors. The National Health Science Research Committee of Malawi granted permission to use this programmatic data to perform the analysis. No patient identifiers were kept in the datasets analysed.

## Results

### Patient characteristics

**Figure 1:**
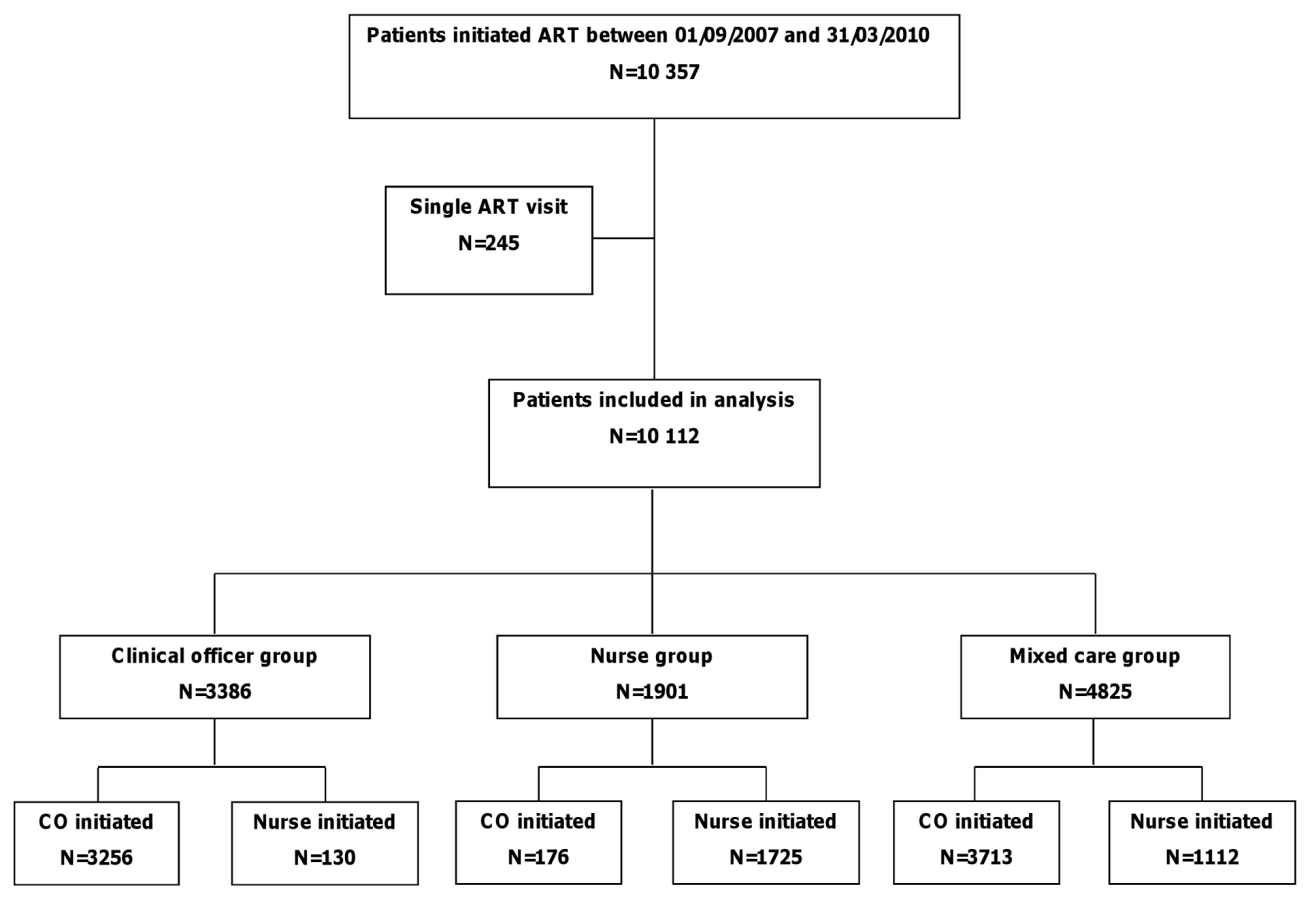
Study profile.

A total of 10,357 adult patients were initiated on ART during the study period, with 245 excluded from this analysis because they only had one ART visit (Figure 1). Of the 10,112 included patients, 3,386 (33.5%) were in the clinical officer group, 1901 (18.8%) in the nurse care group, and 4,825 (47.7%) in the mixed care group. Twenty-three percent of patients included in the mixed care group were initially assessed and started on ART by nurses (n=1,112). Collectively, patients contributed 14,012 person-years of follow-up.

At ART start, patient median age was 35.1 years, and 63.7% of patients were women (Table 1). Ninety-two percent received stavudine-containing therapy, 94.8% had no history of ART and 41.5% were in clinical stage 3 or 4. The proportion of patients with severe HIV disease at ART start was higher in the clinical officer group than in the nurse and mixed groups (28.1% compared to 3.8% and 11.0%, respectively, were in clinical stage 4; and 35.4% compared to 15.4% and 25.9%, respectively, had BMI <18.5 kg/m^2^). Patients in the nurse group had higher median CD4 counts than patients in the clinical officer or mixed care groups (195 [147-234, n=1823], compared to 147 [69-228; n=3058] and 182.0 [113-233; n=4562] cells/µL, respectively). The number of patients followed by nurses and in the mixed care group greatly increased over time, from 170 initiated in 2007 to 976 initiated in 2009, and from 709 to 1677, respectively.

**Table 1:** Patient characteristics at ART initiation by type of health care provider.

**Characteristics**	**Nurse group**		**Mixed care group**				**Clinical officer group**		**Total**
	**N=1901**		**Nurse initiated**	**CO initiated**	**Total**		**N=3386**		**N=10112**
			**N=1112**	**N=3713**	**N=4825**				
**Women, n (%**)	1263 (66.4)		719 (64.7)	2557 (68.9)	3276 (67.9)		1905 (56.3)		6444 (63.7)
**Median age, years [IQR**]	35.1 [29.4-43.1]		35.8 [30.1-44.1]	34.5 [29.1-42.2]	34.9 [29.2-43.0]		35.2 [30.0-43.2]		35.1 [29.4-43.1]
**BMI <18.5 kg/m^2^, n (%**)	293 (15.4)		265 (23.8)	985 (26.5)	1250 (26.0)		1197 (35.9)		2740 (27.3)
**Clinical stage, n (%**)									
1	797 (44.9)		433 (38.9)	1267 (34.1)	1700 (38.9)		656 (20.7)		3153 (33.9)
2	617 (34.7)		347 (31.2)	726 (19.6)	1073 (24.5)		511 (16.2)		2201 (33.6)
3	295 (16.6)		184 (16.5)	935 (25.2)	1119 (25.6)		1106 (35.0)		2520 (27.0)
4	68 (3.8)		55 (4.9)	425 (11.4)	480 (11.0)		891 (28.1)		1439 (14.5)
Missing	124		93	360	453		222		799
**Median CD4 cell count, cells/µL [IQR**]	195 [147-234]		185 [119-232]	181 [109-233]	182 [113-233]		147 [69-228]		178 [105-232]
Missing	78		53	210	263		328		669
**Year of ART initiation, n (%**)									
2007	170 (8.9)		83 (7.5)	626 (16.9)	709 (14.7)		350 (10.3)		1229 (12.2)
2008	501 (26.4)		360 (32.4)	1778 (47.9)	2138 (44.3)		1260 (37.2)		3899 (38.6)
2009	976 (51.3)		561 (50.5)	1116 (30.1)	1677 (34.8)		1399 (41.3)		4052 (40.1)
2010	254 (13.4)		108 (9.7)	193 (5.2)	301 (6.2)		377 (11.1)		932 (9.2)
**Duration of follow-up, months [IQR**]	17.6 [13.2-24.6]		20.2 [13.6-26.9]	24.8 [17.2-32.0]	24.2 [16.1-31.2]		15.2 [5.2-24.2]		19.8 [13.1-28.1]
**Median number of follow-up visits [IQR**]	11 [9-15]		14 [10-18]	16 [12-20]	15 [11-19]		13 [6-19]		14 [9-19]
**Adherence index, n (%**)									
≥95	978 (51.5)		553 (49.7)	195 (52.9)	2518 (52.2)		1637 (48.4)		5133 (50.8)
80-94	814 (42.8)		465 (41.8)	1590 (42.8)	2055 (42.6)		1185 (35.0)		4054 (40.1)
<80	109 (5.7)		94 (8.5)	158 (4.3)	252 (5.2)		564 (16.7)		925 (9.2)

Note: ART, antiretroviral therapy; BMI, body mass index; IQR, interquartile range.

### Immunological response by type of care provider

**Figure 2:**
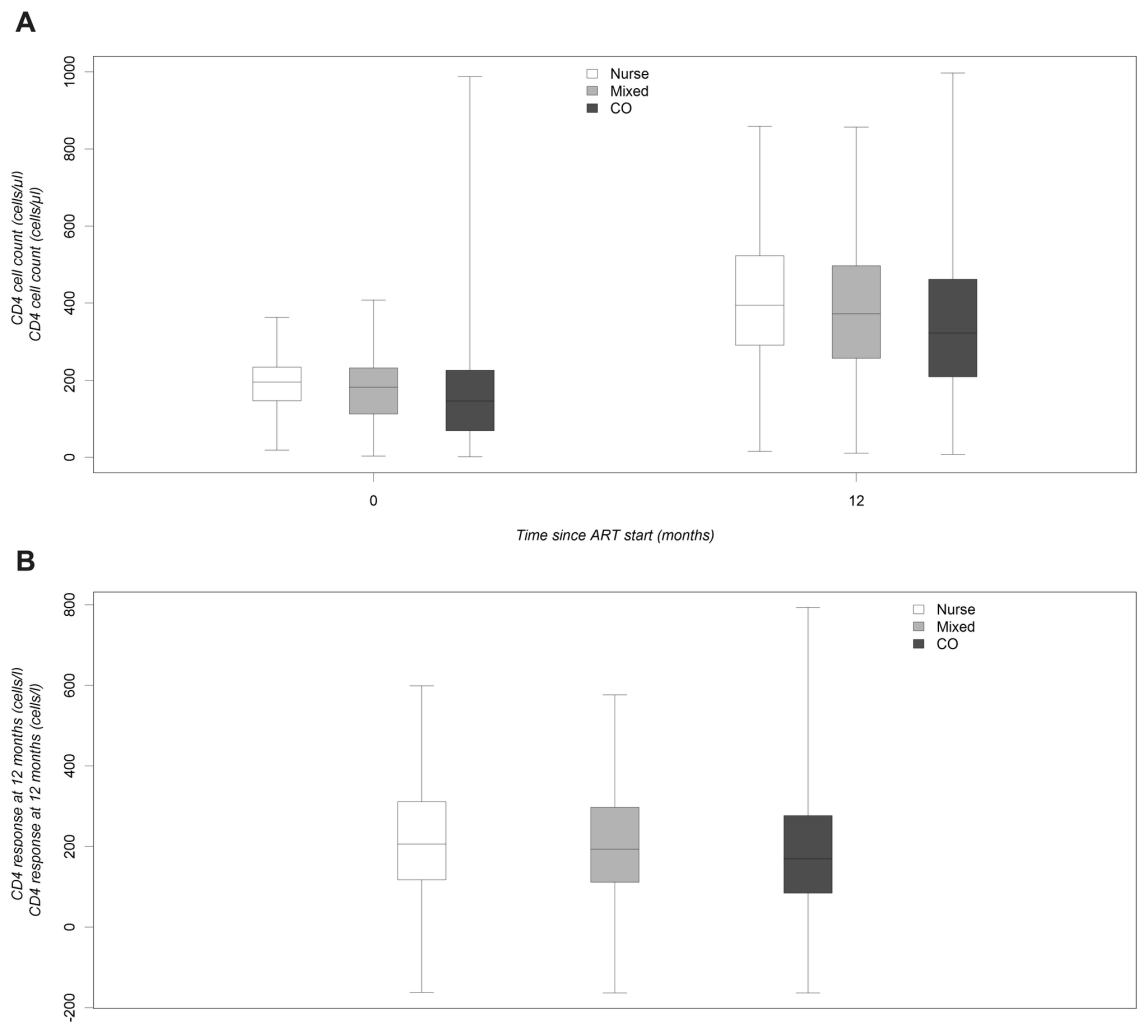
Median absolute CD4 cell count and median CD4 gain 12 months after ART start by type of provider. A) Median absolute CD4 cell count. B) Median CD4 gain 12 months after ART start.

One year after starting ART the median CD4 cell count of patients treated in the nurse group was higher than in the clinical officer and mixed care groups (397 [IQR 293-526; n=1202], 326 [IQR 210-474; n=1994], and 375 [IQR 259-504; n=3502] cells/µL, respectively, Figure 2A). One-year median CD4 count gains were 207 [IQR 118-317, n=1168] in the nurse group, 168 [IQR 81-278, n=1397] in the clinical officer group, and 195 [IQR 112-301, n=3374] cells/µL in the mixed care group (Figure 2B).

### Program attrition and mortality outcomes by type of care provider

**Figure 3:**
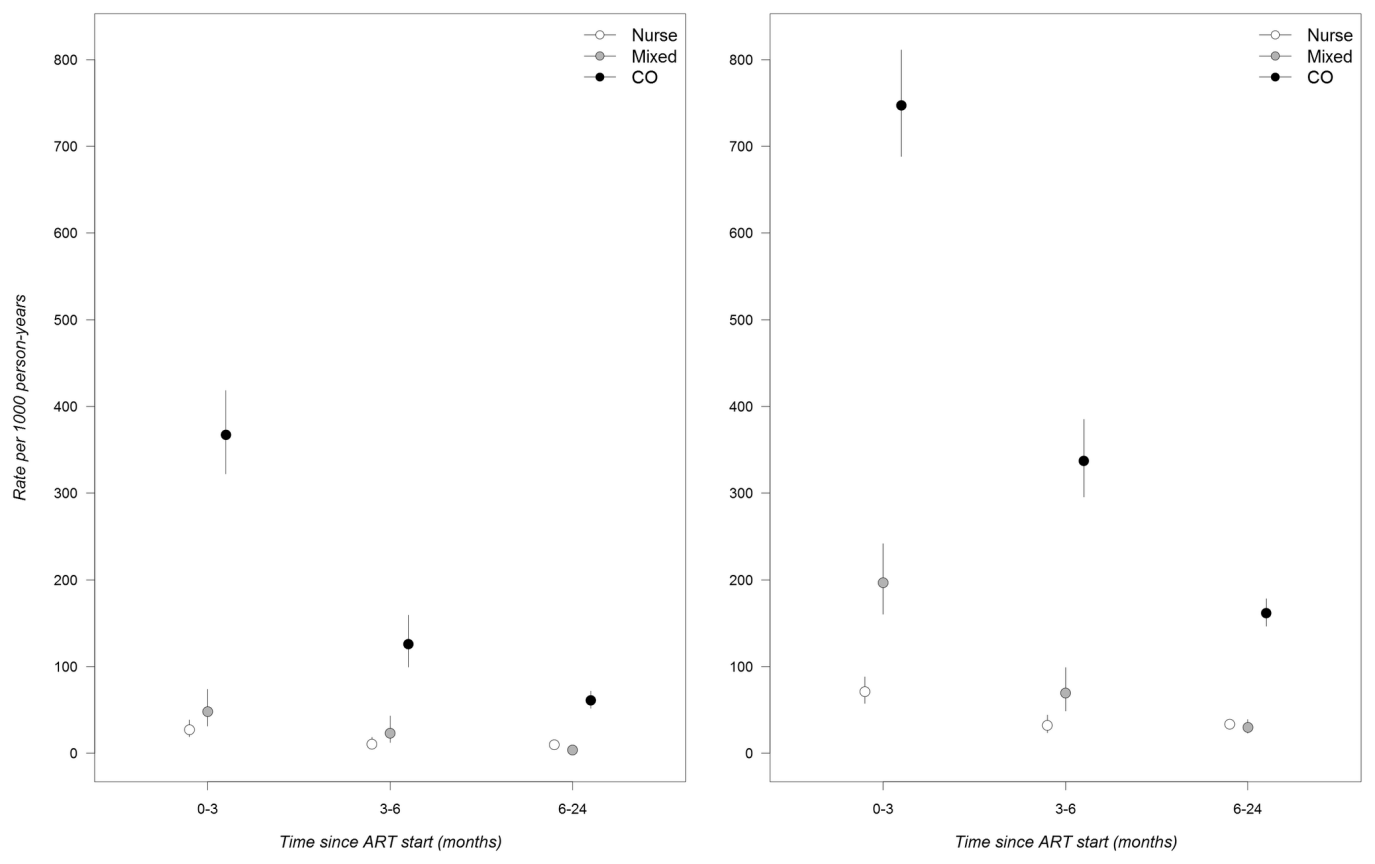
Rates of mortality and program attrition by type of health care provider. A) Mortality. B) Program attrition.

A total of 1,693 lost to follow-up events and 576 deaths were recorded during the study period. The overall 2-year cumulative probability of retention in care was 81.8%. Rates of attrition and mortality were highest during the first 3 months of ART and decreased sharply after 6 months (Figure 3A and 3B). Compared to nurse-managed patients, 2-year program attrition was higher in the clinical officer group and it was lower in the mixed care group (adjusted IRR (aIRR)=3.03, 95%CI 2.56-3.59 for the clinical and aIRR=0.54, 95% CI 0.45-0.65 for the mixed care group; Table 2). Slightly higher estimates were observed when analyses were restricted to patients with less severe disease (aIRR=3.47, 95%CI 2.63-4.58 for the clinical and 0.59, 95%CI 0.43-0.81 for the mixed care group, compared to the nurse group).

**Table 2:** Associations between 2-year program attrition and individual-level factors among all patients and in the subgroup of patients with less severe HIV disease.

**Individual factors**	**All patients**			**Less severe patients**		
	**Crude IRR(95% CI**)	**Adjusted IRR (95% CI**)		**Crude IRR (95% CI**)	**Adjusted IRR (95% CI**)	
**Type of provider**	p<0.001	p<0.001		P<0.001	p<0.001	
Nurse	1	1		1	1	
Mixed	0.60 (0.50-0.73)	0.54 (0.45-0.65)		0.52 (0.38-0.71)	0.59 (0.43-0.81)	
Clinical officer	4.71 (4.02-5.51)	3.03 (2.56-3.59)		3.69 (2.82-4.82)	3.47 (2.63-4.58)	
**Sex**	p<0.001	p<0.001		p<0.001	p<0.001	
Male	1	1		1	1	
Female	0.52 (0.47-0.57)	0.75 (0.68-0.82)		0.55 (0.44-0.69)	0.64 (0.51-0.81)	
**Initial BMI, kg/m^2^**	p<0.001	p<0.001		p=0.62	p=0.43	
<18.5	1	1				
18.5-24.99	0.42 (0.38-0.46)	0.56 (0.50-0.62)		1	1	
≥25	0.34 (0.27-0.44)	0.40 (0.31-0.51)		0.91 (0.63-1.33)	0.86 (0.59-1.26)	
Unknown	3.30 (2.36-4.61)	1.42 (1.00-1.99)				
**Initial clinical stage**	p<0.001	p<0.001		p=0.005	p=0.03	
1	1	1		1	1	
2	1.43 (1.22-1.68)	1.27 (1.08-1.50)		1.38 (1.11-1.72)	1.27 (1.02-1.59)	
3	2.30 (1.99-2.66)	1.23 (1.06-1.43)				
4	4.17 (3.60-4.82)	1.48 (1.26-1.74)				
Unknown	1.91 (1.57-2.33)	1.40 (1.15-1.72)				
**Initial CD4 count, cells/µL**	p<0.001	p<0.001		p=0.20	p=0.31	
<50	1	1				
50-99	0.60 (0.51-0.72)	0.78 (0.66-0.93)				
100-199	0.42 (0.36-0.48)	0.83 (0.71-0.97)		1	1	
200-249	0.31 (0.26-0.37)	0.71 (0.59-0.85)		0.80 (0.63-1.02)	0.83 (0.65-1.06)	
≥250	0.53 (0.45-0.63)	0.90 (0.75-1.07)		0.88 (0.64-1.19)	0.96 (0.70-1.32)	
Unknown	0.95 (0.79-1.15)	1.07 (0.88-1.29)				
**Year of ART initiation**	p=0.02	p=0.02		p=0.03	p=0.10	
2007	1	1		1	1	
2008	1.06 (0.91-1.24)	1.02 (0.87-1.19)		1.21 (0.81-1.81)	1.00 (0.67-1.50)	
2009	1.10 (0.94-1.28)	0.90 (0.77-1.06)		1.60 (1.08-2.37)	1.30 (0.87-1.94)	
2010	1.40 (1.13-1.74)	0.78 (0.63-0.97)		1.56 (0.90-2.68)	0.90 (0.52-1.57)	
**Adherence index**	p<0.001	p<0.001		p<0.001	p<0.001	
≥95%	1	1		1	1	
80-94%	0.42 (0.37-0.47)	0.39 (0.35-0.45)		0.50 (0.38-0.66)	0.49 (0.38-0.65)	
<80%	3.62 (3.22-4.06)	1.84 (1.63-2.07)		4.46 (3.39-5.87)	2.84 (2.14-3.76)	
**Period of follow-up, months**	p<0.001	p<0.001)		p<0.001	p<0.001	
≤3	1	1		1	1	
4-6	0.41 (0.36-0.47)	0.48 (0.42-0.55)		0.42 (0.30-0.59)	0.46 (0.33-0.64)	
7-24	0.21 (0.19-0.24)	0.28 (0.25-0.31)		0.29 (0.23-0.37)	0.33 (0.26-0.43)	

Note: ART, antiretroviral therapy; BMI, body mass index; CI, confidence interval; IRR, incidence rate ratio.

In secondary analyses, patients initiated on ART by clinical officers in the mixed care group had lower attrition (aIRR=0.36, 95%CI 0.29-0.44) than those in the nurse care group; but no differences in attrition were found between patients initiated on ART by nurses in the mixed care group, and those included in the nurse group (aIRR=1.18, 95%CI 0.95-1.47; Table 3).

**Table 3:** Associations between 2-year program attrition and individual-level factors among all patients and in the subgroup of patients with less severe HIV disease, when differentiation between mixed care patients initiated by nurses or clinical officers was made.

	**All patients**	**Patients with less severe disease**
	**Adjusted IRR (95% CI**)	**Adjusted IRR (95% CI**)
**Type of provider**	p<0.001	p<0.001
Nurse	1	1
Mixed nurse initiated	1.18 (0.95-1.47)	1.13 (0.79-1.61)
Mixed clinical officer initiated	0.36 (0.29-0.44)	0.33 (0.22-0.50)
Clinical officer	2.96 (2.50-3.51)	3.41 (2.59-4.51)

Note: CI, confidence interval; IRR, incidence rate ratio.

Compared to patients treated in the nurse group, those treated in the clinical officer group had higher 2-year mortality (adjusted IRR (aIRR)=5.04, 95%CI 3.56-7.15; Table S3), and patients treated in the mixed care group did not significantly differ (aIRR=0.72, 95% CI 0.49-1.06). Slightly higher estimates were also observed when analyses were restricted to patients with less severe disease (aIRR=5.74, 95%CI 3.23-10.19 for the clinical officer and 0.84, 95%CI 0.45-1.59 for the mixed care group, compared to the nurse group). Restriction of analyses to patients with complete case data did not change the results for mortality or program attrition.

### Other associations with program attrition and mortality

Women had lower risk of program attrition compared to men (aIRR=0.75, 95%CI 0.68-0.82), and the risk of attrition increased in patients with more advanced HIV disease at ART start (aIRR=1.27, 95%CI 1.08-1.50 for clinical stage 2 and aIRR=1.48, 95%CI 1.26-174 for stage 4, compared to stage 1; Table 2). The risk of program attrition decreased with higher BMI at the start of ART (aIRR=0.40, 95%CI 0.31-0.51 for ≥25.0 compared to <18.5 kg/m^2^), and with longer duration of ART follow-up (from aIRR=0.48, 95%CI 0.42-0.55 for the 3-6 month period, to aIRR=0.28, 95%CI 0.25-0.31 for the 6-24 month period, compared to ≤3 months). It was lower in patients with higher baseline CD4 cell counts (aIRR=0.83, 95%CI 0.71-0.97 for the 100-199 group, aIRR=0.71, 95%CI 0.59-0.85 for the 200-249 group, and aIRR=0.90, 95%CI 0.75-1.07 for the ≥250 group, compared to <50 cells/µL), and higher in patients with lower adherence index (aIRR=1.84, 95%CI 1.63-2.07 for <80% compared to ≥95%). Estimates from analyses restricted to the group of patients with less severe disease were generally higher, and the association with BMI was no longer significant.

Similar to our findings on program attrition, the risk of mortality was lower in women than in men (aIRR=0.61, 95%CI 0.52-0.72), in patients with higher initial BMI (aIRR=0.14, 95%CI 0.07-0.26 for ≥25 compared to <18.5 kg/m^2^), and in those who started therapy in later time periods (aIRR=0.70, 95%CI 0.48-1.01; Table S3). It was also higher in patients with advanced clinical disease at ART start (aIRR=1.53, 95%CI 1.16-2.02 for clinical stage 4 compared to stage 1), and decreased with higher initial CD4 count levels (from aIRR=0.73, 95%CI 0.55-0.97 for the 50-99 group, to aIRR=0.67, 95%CI 0.49-0.91 for the 200-249 group, compared to <50 cells/µL). Restriction of analyses to patients with complete case data did not change the results for mortality or program attrition (Table S4). Results from analyses based on patients with 3 months or more of follow-up were consistent with findings of the primary analysis (Table S5).

## Discussion

While the deployment of non-physicians for the provision of HIV services is widespread, few data exist on the effectiveness of task-shifting and task-sharing for scaling-up of services in non-research rural settings. The present study provides evidence that it is feasible to achieve high retention rates in large-scale decentralized non-physician driven HIV programs in rural Africa, in settings characterized by a fragile health system and limited diagnostic capacity. The implementation of a mixed care model where nurses and clinical officers refer and back-refer patients according to their evolving clinical status was associated with reduced program attrition, compared to management primarily by either nurses only or clinical officers only. However, findings of secondary analyses that distinguished between the type of provider who initiated ART in the mixed care group, suggested that patients initiated on therapy by clinical officers might have better treatment outcomes than those managed by nurses, and that further efforts to improve training and supervision of non-physician health providers are required.

The overall 2-year cumulative retention in HIV care in the program was 81%. The findings of this study are in line with results from evaluations of treatment outcomes associated with task-shifting [16-18,25,26]. Most previous studies evaluated models of care based on task-shifting of ART care monitoring, that is down-referral of clinically stable patients after ART initiation. Defining which types of health care workers can provide HIV care safely and identifying what models of care facilitate expanded access are imperative to scaling up treatment so it reaches the millions of HIV-infected people still in need [27,28].

Several studies specifically evaluated treatment outcomes of patients initiated and managed by nurses. In the CIPRA-SA randomized trial including 812 adults, Sanne and colleagues reported the non-inferiority of nurse ART monitoring compared to physician-monitoring implemented in South African primary health clinics [18]. The composite endpoint of treatment-limiting events included mortality, viral failure, toxicity and clinical appointment compliance. More than 40% of patients in each arm experienced an endpoint. In Rwanda, Shumbusho et al. reviewed medical records from 1076 ART-naïve uncomplicated adult patients treated in a nurse-centered care program implemented in three primary health centers [17]. Nurses closely supervised by physicians correctly initiated patients on ART. Patient retention at facility level was 80.5% at 2 years. In Lesotho, Cohen et al. reported that, 2 years after ART start in a rural nurse-driven program, 77% of adults remained in HIV care in a rural nurse-driven program.

Most recently Fairall and colleagues presented the results of a cluster-randomized trial, the STRETCH study, and showed that shifting responsibility of ART initiation from physicians to nurses did not decrease patient survival for those already receiving ART; and that the proportion of patients already on ART with viral suppression was not significantly different in the two groups [29]. While nurses had little trouble assuming responsibilities shifted to them, there was limited evidence of improved ART initiation or reduced mortality in the cohort with nurses initiating.

In this study the nurse group initiated 1 in 3 patients on ART. Sharing responsibilities between all available health care workers allowed for more patients to initiate ART from 2,700 in 2006 to 4,389 in 2008. The nurse and mixed care group had better outcomes than the clinical officer group, but patients in the mixed care group who were initiated by clinical officers had lower attrition than those included in the nurse group. There are several possible reasons as to why the nurse and mixed care group had better outcomes compared to the clinical officer group. First, severe and complicated patients were treated by or referred to clinical officers, so that the sickest patients with the highest risk of mortality were more likely to be included in the clinical officer group. Second, while the high mortality and attrition estimates observed in this group were not unexpected given the severity of disease at ART start, the difference in estimates with respect to the other groups was extremely high, even when rates were compared within the subgroup of patients with less severe HIV disease, or within the subgroup of patients who were in care after the first three months of ART. It is therefore possible that incomplete adjustment by factors not available for this analysis, and/or misclassification of certain patients with severe HIV disease, partly explains the observed difference. Nevertheless, while nurses or medical assistants might sometimes wrongly stage patients, particularly at ART initiation, misclassification of clinical staging would likely have resulted in underestimation of the differences in poor outcomes between the nurse and the clinical officer groups instead of magnifying them, as non-physicians would have been managing complicated cases.

In our study, we did not evaluate pre-ART nurse management and were therefore not able to ascertain whether nurses correctly identified patients eligible for ART. The secondary analyses, however, suggested that initial evaluation of patients at ART start by clinical officers might be associated with better outcomes and that training and supervision of non-physician health providers need to be strengthened and monitored. Even with the district’s major scale-up of ART care, almost half of the patients included in this analysis had a clinical stage 3 or 4, or were severely immuno-compromised (<100 CD4 count) at ART initiation, highlighting the urgent ongoing need for expanding regional access to HIV testing and treatment. Early mortality is strongly associated with the clinical and immunological status of patients [30,31]. Increasing access to services and increasing the CD4 threshold at ART initiation will reduce early mortality [15,32]. Furthermore, earlier start of ART allows further simplification of medical follow-up in settings such as Malawi and reduces the incidence of clinical complications.

Interpreting the results of this evaluation should be done in the light of several limitations. First, patients were not randomly assigned to the specific provider/strategy-of-care groups and patients with more severe disease were primarily treated by clinical officers. However, results from sensitivity analyses restricted to patients with 3 months or more of follow-up were consistent with those from the primary analysis, showing higher attrition in the clinical officer group. Second, although strategies of care evolved over time, the scale-up and decentralization of the program started in 2003 and the analysis period began six years after the start of ART provision in the program, and over two years after nurses began HIV care management training and delivery. In addition, multivariable analyses were adjusted for the effect of the year of ART start. An either- or scenario (exclusive provision of care by nurse or by clinical officer) is nevertheless not a realistic approach in the sub-Saharan context due to limited supply of all health care workers.

Third, this study did not take into account differences in additional patient workload or responsibilities that nurses and clinical officers routinely assume. These tasks include provision of antenatal, hospital and pediatric care, management of long-term ART experienced patients, phlebotomy, or dispensing of first-line alternative and second-line ART regimens.

Fourth, the present evaluation was based on the analysis of an electronic HIV monitoring database. This type of data is prone to errors related to inaccuracy and incompleteness of information, specifically for laboratory measurements. Nevertheless, ongoing rigorous data management procedures, including monthly review and cross-verification of 10% of medical files, have been in place for several years which minimized data entry errors. Additionally, we compared outcomes using sensitivity analysis with complete case data and found similar results. Finally, as reported in a previous study in the same setting, mortality is underestimated due to incomplete death ascertainment in the program; up to 54% of adults started on ART and lost to follow-up might have actually died [33]. Nonetheless, the choice of attrition as the primary study outcome and the consistency of results found when various sensitivity analyses were performed, increases the robustness of our findings.

## Conclusion

Our evaluation contributes to the growing evidence that nurses can safely initiate ART and clinically monitor adult patients, provided that adequate training, supervision and referral criteria are implemented. Shifting responsibilities to lower health cadres for HIV care delivery seems to be a good interim solution to the healthcare worker crisis in highly prevalent HIV African countries. However, completely shifting responsibility of ART care delivery to nurses or other non-physician providers requires caution. There is a need to develop and evaluate context-models of care that rely less on traditional clinical care providers. As demonstrated through the lower attrition in the mixed care group, task-sharing can be a more realistic approach to ensure that patients are treated timely and accessibly.

## Supporting Information

Table S1 **Distribution of tasks and responsibilities between different types of providers in the Chiradzulu HIV programme.**
(DOCX)

Table S2 **Nurse training Module objectives and validation criteria.**
(DOCX)

Table S3 **Associations between mortality and risk factors among all patients and in the subgroup of patients with less severe HIV disease.**
(DOCX)

Table S4 **Associations between program retention and mortality and risk factors among all patients and in the subgroup of patients with less severe HIV disease [Complete case data analysis].**
(DOCX)

Table S5 **Association between program attrition and risk factors among patients who were receiving HIV care after 3 months of follow-up.**
(DOCX)
