# Supplementary material for: Task-Sharing of HIV Care and ART Initiation: Evaluation of a Mixed-Care Non-Physician Provider Model for ART Delivery in Rural Malawi
Source: PLoS One. 2013 Sep 16;8(9):e74090. doi: 10.1371/journal.pone.0074090 (PMC3774791; doi:10.1371/journal.pone.0074090)
Supplement: Table S1 — Distribution of tasks and responsibilities between different types of providers in the Chiradzulu HIV programme. (DOCX) [file pone.0074090.s001.docx]

**Table S1. Distribution of tasks and responsibilities between different types of providers in the Chiradzulu HIV programme**

|  | **Clinical Officer** | **Nurse** | **Medical Assistant** | **Counselor** | **Peer Counselor** | **Community Health Worker** |
| --- | --- | --- | --- | --- | --- | --- |
| HIV rapid diagnostic test |  |  |  |  |  | **x** |
| Initial physical exam and staging | **x** | **x** | **x** |  |  |  |
| Assessment of ART eligibility | **x** | **x** | **x** |  |  |  |
| Follow-up of non-eligible patients | **x** | **x** | **x** |  |  |  |
| Pediatric follow-up | **x** |  |  |  |  |  |
| Prevention of mother to child transmission of HIV infection |  | **x** |  |  |  |  |
| ART care, requesting laboratory tests | **x** | **x** | **x** |  |  |  |
| Interpretation of laboratory tests results | **x** | **x** | **x** |  |  |  |
| ART initiation and follow-up of uncomplicated cases | **x** | **x** | **x** |  |  |  |
| ART initiation and follow-up of complicated cases* | **x** |  |  |  |  |  |
| Adherence counseling |  |  |  | **x** | **x** |  |
| Tracing of default patients |  |  |  |  |  | **x** |
| ART refill |  | **x** |  |  |  | **x** |
| Register keeping, reporting |  | **x** | **x** | **x** |  | **x** |
| Training, mentoring, supervision | **x** | **x** |  |  |  |  |

**Complicated cases are patients with* suspicion of tuberculosis or treatment failure, patients with Kaposi’s sarcoma, those receiving second line ART and pediatric patients (<15 years old).
