# Supplementary material for: Task-Sharing of HIV Care and ART Initiation: Evaluation of a Mixed-Care Non-Physician Provider Model for ART Delivery in Rural Malawi
Source: PLoS One. 2013 Sep 16;8(9):e74090. doi: 10.1371/journal.pone.0074090 (PMC3774791; doi:10.1371/journal.pone.0074090)
Supplement: Table S2 — Nurse training Module objectives and validation criteria. (DOCX) [file pone.0074090.s002.docx]

**Table S2. Nurse training Module objectives and validation criteria**

| **Nurse Training Modules** | **Curriculum objectives** |
| --- | --- |
| Model 1 | 1. Diagnose and treat all WHO clinical stage 2 and 3 infections 2. Refer patients when necessary |
| Model 2 | 1. Gain knowledge and attain skills for adherence counseling (1^st^, 2^nd^ and follow up counseling sessions) 2. Refer patients to the adherence counselor if they cannot manage them 3. Accurately dispense ARVs to all patients (start, follow-up, pediatric dosage, second line, etc) 4. Consult stable patients and refer to clinician when necessary 5. Record and account for ARVs dispensed |
| Model 3 | 1. Stage new patients 2. Interpret CD4 count results 3. Teach patients about ART use 4. Assess readiness of ARV initiation, including acceptance of disease and commitment to life-time treatment |
| Validation and supervision | Ongoing supervision and refresher training is carried out by a team of 1 nurse and 1 clinical officer. The Malawian Ministry of Health implements ART certification training with a written test. |
