# Supplementary material for: Task-Sharing of HIV Care and ART Initiation: Evaluation of a Mixed-Care Non-Physician Provider Model for ART Delivery in Rural Malawi
Source: PLoS One. 2013 Sep 16;8(9):e74090. doi: 10.1371/journal.pone.0074090 (PMC3774791; doi:10.1371/journal.pone.0074090)
Supplement: Table S3 — Associations between mortality and risk factors among all patients and in the subgroup of patients with less severe HIV disease. (DOCX) [file pone.0074090.s003.docx]

**Table S3. Associations between 2-year mortality and individual-level factors among all patients and in the subgroup of patients with less severe HIV disease**

| **Factors** | **All patients** | |  | **Less severe patients** | |  |
| --- | --- | --- | --- | --- | --- | --- |
|  | **Crude IRR (95% CI)** | **Adjusted IRR (95% CI)** | | **Crude IRR (95% CI)** | **Adjusted IRR (95% CI)** | |
| **Type of provider** | p<0.001 | p<0.001 | | p<0.001 | p<0.001 | |
| Nurse | 1 | 1 | | 1 | 1 | |
| Mixed | 0.88 (0.60-1.28) | 0.72 (0.49-1.06) | | 0.76 (0.41-1.41) | 0.84 (0.45-1.59) | |
| Clinical officer | 8.02 (5.76-11.18) | 5.04 (3.56-7.15) | | 5.15 (2.94-9.01) | 5.74 (3.23-10.19) | |
| **Sex** | p<0.001 | p<0.001 | | p<0.001 | p=0.002 | |
| Male | 1 | 1 | | 1 | 1 | |
| Female | 0.41 (0.35-0.48) | 0.61 (0.52-0.72) | | 0.43 (0.28-0.66) | 0.49 (0.32-0.76) | |
| **Initial BMI, kg/m^2^** | p<0.001 | p<0.001 | | p=0.06 | p=0.05 | |
| <18.5 | 1 | 1 | |  |  | |
| 18.5-24.99 | 0.27 (0.23-0.32) | 0.37 (0.31-0.44) | | 1 | 1 | |
| ≥25 | 0.11 (0.06-0.21) | 0.14 (0.07-0.26) | | 0.43 (0.16-1.18) | 0.41 (0.15-1.13) | |
| Missing | 2.33 (1.28-4.24) | 0.98 (0.53-1.81) | |  |  | |
| **Initial clinical stage** | p<0.001 | p=0.02 | | p<0.001 | p=0.003 | |
| 1 | 1 | 1 | | 1 | 1 | |
| 2 | 1.56 (1.16-2.09) | 1.32 (0.98-1.77) | | 2.16 (1.41-3.32) | 1.94 (1.26-2.99) | |
| 3 | 2.67 (2.06-3.45) | 1.15 (0.88-1.51) | |  |  | |
| 4 | 5.40 (4.18-6.98) | 1.53 (1.16-2.02) | |  |  | |
| Missing | 2.05 (1.44-2.92) | 1.32 (0.92-1.89) | |  |  | |
| **Initial CD4 count, cells/µL** | p<0.001 | p=0.04 | | p=0.18 | p=0.44 | |
| <50 | 1 | 1 | |  |  | |
| 50-99 | 0.52 (0.39-0.69) | 0.73 (0.55-0.97) | |  |  | |
| 100-199 | 0.37 (0.29-0.47) | 0.87 (0.68-1.12) | | 1 | 1 | |
| 200-249 | 0.23 (0.17-0.30) | 0.67 (0.49-0.91) | | 0.71 (0.44-1.14) | 0.77 (0.48-1.23) | |
| ≥250 | 0.43 (0.32-0.57) | 0.92 (0.68-1.24) | | 0.61 (0.31-1.17) | 0.72 (0.37-1.42) | |
| Missing | 0.75 (0.54-1.03) | 1.03 (0.74-1.43) | |  |  | |
| **Year of ART initiation** | p=0.14 | p=0.002 | | p=0.70 | p=0.38 | |
| 2007 | 1 | 1 | | 1 | 1 | |
| 2008 | 1.02 (0.79-1.30) | 1.02 (0.79-1.31) | | 0.92 (0.48-1.78) | 0.87 (0.45-1.69) | |
| 2009 | 0.87 (0.67-1.12) | 0.73 (0.57-0.95) | | 0.94 (0.49-1.81) | 0.92 (0.47-1.80) | |
| 2010 | 1.23 (0.85-1.77) | 0.70 (0.48-1.01) | | 0.51 (0.14-1.80) | 0.38 (0.11-1.36) | |
| **Adherence index** | p<0.001 | p<0.001 | | p=0.08 | p=0.10 | |
| ≥95% | 1 | 1 | | 1 | 1 | |
| 80-94% | 0.35 (0.28-0.43) | 0.33 (0.27-0.41) | | 0.62 (0.39-0.98) | 0.61 (0.38-0.97) | |
| <80% | 2.28 (1.84-2.84) | 1.04 (0.83-1.31) | | 1.24 (0.53-2.88) | 0.71 (0.30-1.68) | |
| **Period of follow-up, months** | p<0.001 | p<0.001 | | p<0.001 | p<0.001 | |
| ≤3 | 1 | 1 | | 1 | 1 | |
| 4-6 | 0.35 (0.28-0.45) | 0.42 (0.33-0.54) | | 0.33 (0.17-0.66) | 0.36 (0.18-0.70) | |
| 7-24 | 0.18 (0.15-0.22) | 0.24 (0.20-0.28) | | 0.27 (0.17-0.43) | 0.29 (0.18-0.46) | |

Note: ART, antiretroviral therapy; BMI, body mass index; CI, confidence interval; IRR, incidence rate ratio.
