# Supplementary material for: Task-Sharing of HIV Care and ART Initiation: Evaluation of a Mixed-Care Non-Physician Provider Model for ART Delivery in Rural Malawi
Source: PLoS One. 2013 Sep 16;8(9):e74090. doi: 10.1371/journal.pone.0074090 (PMC3774791; doi:10.1371/journal.pone.0074090)
Supplement: Table S4 — Associations between program retention and mortality and risk factors among all patients and in the subgroup of patients with less severe HIV disease [Complete case data analysis]. (DOCX) [file pone.0074090.s004.docx]

**Table S4. Associations between 2-year program attrition and mortality and individual-level factors [Complete case data]**

| **Factors** | **Program attrition** | **Mortality** |
| --- | --- | --- |
|  | **Adjusted IRR (95% CI)** | **Adjusted IRR (95% CI)** |
| **Type of provider** | p<0.001 | P<0.001 |
| Nurse | 1 | 1 |
| Mixed | 0.52 (0.42-0.63) | 0.73 (0.48-1.11) |
| Clinical officer | 3.11 (2.58-3.76) | 5.33 (3.64-7.82) |
| **Sex** | p<0.001 | p<0.001 |
| Male | 1 | 1 |
| Female | 0.76 (0.69-0.85) | 0.62 (0.51-0.74) |
| **Initial BMI, kg/m^2^** | p<0.001 | p<0.001 |
| <18.5 | 1 | 1 |
| 18.5-24.99 | 0.57 (0.51-0.64) | 0.39 (0.32-0.48) |
| ≥25 | 0.36 (0.27-0.49) | 0.14 (0.007-0.29) |
| **Initial clinical stage** | p<0.001 | p=0.008 |
| 1 | 1 | 1 |
| 2 | 1.28 (1.08-1.52) | 1.34 (0.99-1.81) |
| 3 | 1.19 (1.01-1.40) | 1.13 (0.85-1.51) |
| 4 | 1.55 (1.31-1.84) | 1.55 (1.16-2.08) |
| **Initial CD4 count, cells/µL** | p=0.02 | p=0.10 |
| <50 | 1 | 1 |
| 50-99 | 0.78 (0.65-0.93) | 0.78 (0.57-1.05) |
| 100-199 | 0.86 (0.73-1.02) | 0.97 (0.74-1.27) |
| 200-249 | 0.74 (0.61-0.90) | 0.75 (0.54-1.04) |
| ≥250 | 0.86 (0.71-1.04) | 1.05 (0.76-1.44) |
| **Year of ART initiation** | p=0.02 | p=0.01 |
| 2007 | 1 | 1 |
| 2008 | 0.98 ( 0.83-1.17) | 0.94 (0.72-1.25) |
| 2009 | 0.89 (0.74-1.05) | 0.73 (0.55-0.97) |
| 2010 | 0.73 (0.58-0.93) | 0.62 (0.41-0.94) |
| **Adherence Index** | p<0.001 | p<0.001 |
| ≥95% | 1 | 1 |
| 80-94% | 0.40 (0.35-0.46) | 0.34 (0.27-0.43) |
| <80% | 2.01 (1.76-2.30) | 1.07 (0.83-1.39) |
| **Period of follow-up, months** | p<0.001 | p<0.001 |
| ≤3 | 1 | 1 |
| 4-6 | 0.49 (0.42-0.57) | 0.42 (0.32-0.54) |
| 7-24 | 0.28 (0.25-0.32) | 0.25 (0.20-0.30) |

Note: ART, antiretroviral therapy; BMI, body mass index; CI, confidence interval; IRR, incidence rate ratio.
