# Supplementary material for: Task-Sharing of HIV Care and ART Initiation: Evaluation of a Mixed-Care Non-Physician Provider Model for ART Delivery in Rural Malawi
Source: PLoS One. 2013 Sep 16;8(9):e74090. doi: 10.1371/journal.pone.0074090 (PMC3774791; doi:10.1371/journal.pone.0074090)
Supplement: Table S5 — Association between program attrition and risk factors among patients who were receiving HIV care after 3 months of follow-up. (DOCX) [file pone.0074090.s005.docx]

**Table S5. Association between program attrition and risk factors among patients who were receiving HIV care after 3 months of follow-up**

Note: ART, antiretroviral therapy; BMI, body mass index; CI, confidence interval; IRR, incidence rate ratio.
